# Supplementary material for: Profiling Patients by Intensity of Nursing Care: An Operative Approach Using Machine Learning
Source: J Pers Med. 2020 Dec 14;10(4):279. doi: 10.3390/jpm10040279 (PMC7768500; doi:10.3390/jpm10040279)
Supplement: Supplementary file 1 [file jpm-10-00279-s001.pdf]

Supplementary material

Supplementary Materials, Figure 1 Distribution of silhouette width by cluster in the CLARA algorithm. Green dotted lines comprises the best possible cluster; blue dotted lines, uncertain cluster; and red, possibly wrong cluster.

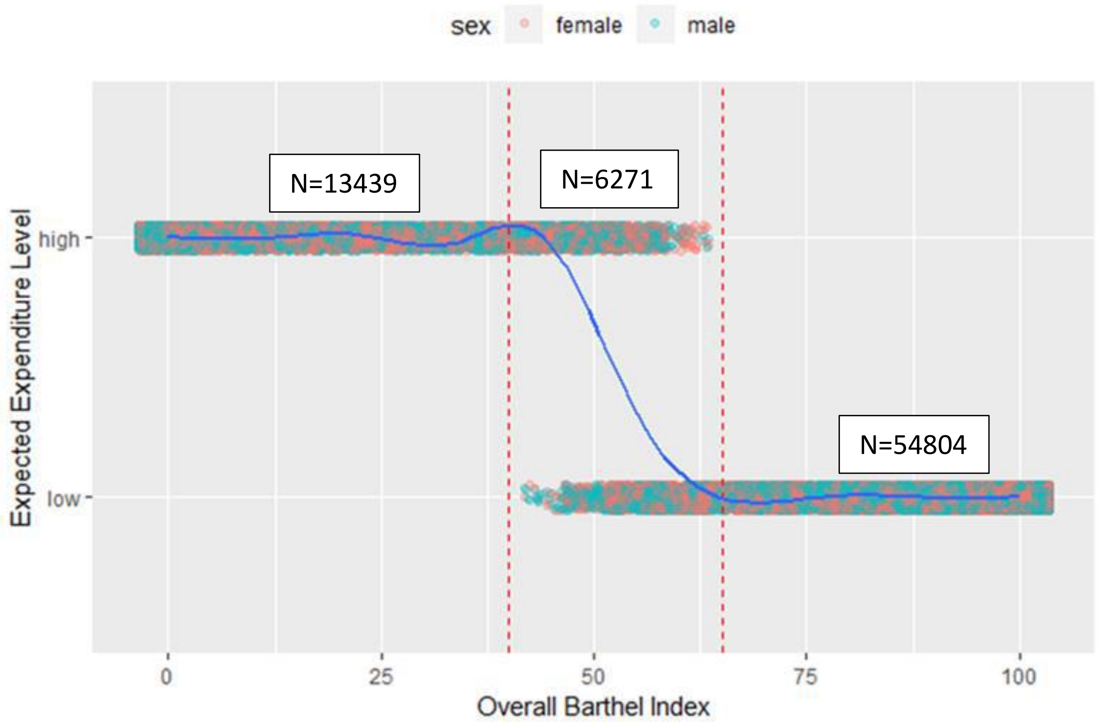

Supplementary Materials,Table S1 Predictive performances of the two-step algorithm over Group 1 (B1<45) and Group 2 (BI>65), the gold standard, and in the overall sample. Measures of performances are evaluated using the data provided by the validation part of the 10-fold cross-validation. In the third column, results for the overall algorithm.

	**Sensitivity**	**Specificity**	**Accuracy**	**F1**
**Group 1 (BI <45), Group 2 (BI>65)**	0.99	0.99	0.99	0.97
**Gold standard**	0.62	0.71	0.67	0.64
